# Lack of treatment-related mortality definitions in clinical trials of children, adolescents and young adults with lymphomas, solid tumors and brain tumors: a systematic review

**DOI:** 10.1186/1471-2407-14-612

**Published:** 2014-08-26

**Authors:** Thai Hoa Tran, Michelle Lee, Sarah Alexander, Paul Gibson, Ute Bartels, Donna L Johnston, Carol Portwine, Marianna Silva, Jason D Pole, Lillian Sung

**Affiliations:** Division of Haematology/Oncology, The Hospital for Sick Children, 555 University Ave, Toronto, Ontario M5G 1X8 Canada; Child Health Evaluative Sciences, The Hospital for Sick Children, Peter Gilgan Centre for Research and Learning, 686 Bay St, Toronto, Ontario M5G 0A4 Canada; Haematology/Oncology, Department of Pediatrics, London Health Sciences Centre, PO Box 5010800, Commissioners Rd E, London, N6A 5W9 Canada; Division of Hematology/Oncology, Children’s Hospital of Eastern Ontario, 401 Smyth Rd, Ottawa, Ontario K1H 8L1 Canada; Hematology/Oncology, McMaster Children’s Hospital, 1200 Main St W, Hamilton, Ontario L8N 3Z5 Canada; Hematology/Oncology, Cancer Centre of Southeastern Ontario, 25 King St W, Kingston, Ontario K7L 5P9 Canada; Pediatric Oncology Group of Ontario, Dalla Lana School of Public Health, University of Toronto, 155 College St, Toronto, Ontario M5T 3M7 Canada

**Keywords:** Treatment-related mortality, Toxic death, Cancer, Pediatric, Adolescents, Young adults, Systematic review

## Abstract

**Background:**

There is a lack of standardized definition for treatment-related mortality (TRM), which represents an important endpoint in cancer. Our objective was to describe TRM definitions used in studies of children, adolescents and young adults with lymphomas, solid tumors and brain tumors.

**Methods:**

We conducted a systematic review of studies enrolling children, adolescents and young adults with lymphomas, solid tumors and brain tumors in which an anti-cancer intervention was randomized, or all study designs in which TRM was a primary or secondary outcome. We searched Ovid MEDLINE, EMBASE and Evidence-Based Medicine Reviews from 1980 to June 2013. Two reviewers evaluated study eligibility and abstracted data.

**Results:**

In total, 67 studies were included and consisted of 62 randomized therapeutic trials and 5 TRM studies. None of the studies (0/67) provided a definition for TRM. Only one randomized trial of rhabdomyosarcoma provided a definition of early death.

**Conclusions:**

We were unable to identify any TRM definitions used in studies of children, adolescents and young adults with lymphomas, solid tumors and brain tumors. Given that a proportion of this patient population may receive intensive treatment, there is an urgent need for consensus-based definitions of TRM for use across clinical trials.

**Electronic supplementary material:**

The online version of this article (doi:10.1186/1471-2407-14-612) contains supplementary material, which is available to authorized users.

## Background

Treatment-related mortality (TRM) is essential information for physicians involved in the care of children, adolescents and young adults with cancer. Outcomes for pediatric cancer have improved remarkably over time and the 5-year overall survival rate for childhood cancers currently exceeds 80% [1]. However, many children with cancer still die and cancer remains the second most common cause of death for North American children and adolescents (after accidents) [1, 2]. As cure rates continue to improve, TRM is predicted to account for a growing proportion of deaths in this population [3].

Describing and identifying predictors of TRM are critically important. Appreciating TRM versus disease-related death is fundamental to understanding the best strategy to improve overall survival. For instance, if TRM is the primary cause of failure for a specific cancer, then the strategy must focus on enhancing supportive care and/or using less toxic therapies [1]. Conversely, if disease progression is the primary cause of death, efforts could be directed towards identification of novel therapies to improve disease control. In addition, correct identification of TRM and disease-related mortality allows for appropriate monitoring of outcomes between trials and over time.

In spite of the critical importance of TRM, epidemiological investigation into TRM characteristics and risk factors has been crippled by the lack of a standardized definition for TRM. We previously published a systematic review of pediatric acute leukemia trials in order to understand the frequency with which TRM has been defined and to describe the utilized definitions. We found most pediatric acute leukemia trials do not describe how TRM was defined and when described, we found great heterogeneity of TRM definitions across trials [4]. In order to comprehensively assess TRM definition across all childhood cancers, we performed a systematic review of randomized therapeutic trials of populations omitted in our previous review, namely trials involving children, adolescents and young adults being treated for lymphomas, solid tumors and brain tumors. Our objectives were (1) to determine the frequency with which TRM has been defined; and (2) to describe the utilized TRM definitions among studies of lymphomas, solid tumors and brain tumors.

## Methods

### Data sources and searches

We developed a protocol for review and followed PRISMA (Preferred Reporting Items for Systematic reviews and Meta-Analysis) guidelines [5]. We performed comprehensive searches for relevant trials using Ovid MEDLINE and EMBASE from 1980 to June 2013 and Evidence-Based Medicine (EBM) reviews-Cochrane Central Register of Controlled Trials from 1980 to the second quarter of 2013, without restriction of language or publication status. The search strategy included the following medical subject heading terms: “neoplasms”, “drug therapy”, “combined modality therapy”, “treatment-related mortality”, “randomized controlled trial”, “treatment mortality”. We also included multiple synonyms, abbreviations, and related keywords for each of these terms. The search strategy can be found in Additional file 1. We focused on two types of studies, namely: (1) trials in lymphoma, solid tumor or brain tumor patients in which an anti-cancer intervention was applied in a randomized fashion; and (2) any type of study with TRM as a primary or secondary outcome. We also examined the reference lists of retrieved original and review articles. As this study was a systematic review of primary studies, no ethical approval was required.

### Study selection

Inclusion and exclusion criteria were defined a priori. Randomized therapeutic trials were included if: (1) study was comprised solely of children, adolescents or young adults (age defined by each study but generally included patients up to 30 years of age); (2) population consisted of newly diagnosed lymphoma, solid tumor or brain tumor patients (ie. not relapsed); (3) there was randomization of anti-cancer treatment (to ensure the study was conducted prospectively) including radiotherapy and surgery alone; and (4) treatment did not consist of solely of hematopoietic stem cell transplantation (HSCT). Exclusion criteria were as follows: (1) no randomized intervention; (2) randomized intervention related to leukemia/myelodysplastic syndrome (MDS) treatment; (3) study included adult subjects above the age of 30; (4) report contained results of more than one, separate randomized controlled trial (RCT) (ie. a review); (5) phase 1 trial; (6) duplicate publication; (7) published before 1990; (8) non-English publication; and (9) abstract form only. When duplicate studies were identified, the publication with the longest follow-up was chosen. For studies in which TRM was an outcome, they were included if: (1) study was comprised solely of children, adolescents or young adults up to the age of 30; (2) population consisted of all types of cancers (3) TRM was a primary or secondary outcome; (4) treatment did not consist solely of HSCT. Exclusion criteria were similar to those of therapeutic randomized trials except that randomization was not required.

One reviewer (LS, ML or THT) screened the titles and abstracts of publications identified by the search strategy. Articles thought to be potentially eligible were retrieved in full and each of these articles was independently assessed for eligibility by two reviewers (ML and THT). Final inclusion of studies into the systematic review was by agreement of both reviewers and discrepancies were resolved by consensus. The reviewers were not blinded to study authors or outcomes. Data abstraction was performed by two reviewers (ML and THT) using standardized data collection form.

### Outcome measures and definitions

The outcome of interest was the presence of TRM definitions in studies of children, adolescents and young adults with lymphomas, solid tumors and brain tumors and if present, to describe how these definitions were defined. We also looked at the reporting of the following variables of interest when considering TRM: whether TRM over entire treatment course was described, reporting of deaths before starting treatment, deaths after completing treatment, deaths after HSCT and deaths from accidents, suicide or unknown cause.

## Results and discussion

**Figure 1:**
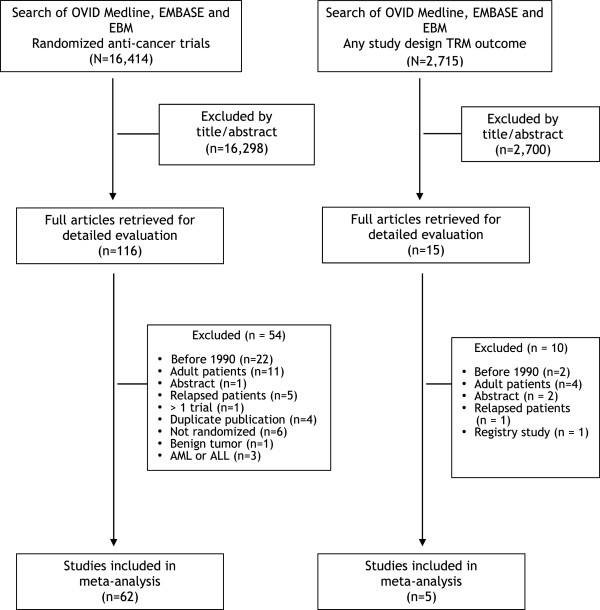
**Flow diagram illustrating flow of studies identified by search strategy and reasons for exclusion.**

Figure 1 illustrates the flow diagram of trial identification and selection. A total of 19,129 titles and abstracts were reviewed; 131 full articles were retrieved for detailed evaluation, and 67 satisfied eligibility criteria to be included in the systematic review. Of these studies, 62 were randomized therapeutic studies and 5 were studies in which TRM was a primary or secondary outcome. Reasons for exclusion are detailed in Figure 1.

Table 1 summarizes the data related to TRM definitions among the two types of studies. None of the therapeutic randomized trials or TRM studies provide a definition of TRM for this population. Of these studies, one study did refer to the concept of early death. This multicenter randomized trial from the International Rhabdomyosarcoma Study Group (IRSG) defined early death as any death occurring within 6 weeks of treatment initiation [6].

**Table 1:** **Summary of TRM reporting in pediatric lymphoma, solid and brain tumor studies**

	Provide definition of TRM	Describe TRM over entire course of treatment	Include deaths before starting chemotherapy	Include deaths after completing chemotherapy	Include deaths after stem cell transplantation	Include accidents, suicide, or unknown
Therapeutic studies N = 62	0	10 (16.1)	11 (17.7)	21 (33.9)	4 (6.1)	9 (14.5)
TRM studies N = 5	0	3 (60.0)	2 (40.0)	4 (80.0)	0	4 (80.0)

Values are n (%).

In addition, 12 therapeutic and 3 TRM studies reported their TRM rate. Thirteen studies evaluated it over the entire treatment course while 2 reported it by phase of therapy (for example; during induction, maintenance or the radiotherapy period). Among therapeutic studies, TRM rates ranged from 0.2% to 7.0% (mean 2.7%) in these patient populations that consisted of neuroblastoma (n = 996), rhabdomyosarcoma (n = 2073), medulloblastoma (n = 364), soft tissue sarcomas (n = 1115), Hodgkin lymphoma (n = 1572), and non-Hodgkin lymphoma (n = 280). Hodgkin lymphoma patients had the lowest TRM rate while the highest TRM rate was reported among neuroblastoma patients. For TRM studies (n = 3), reported TRM rates were much higher, ranging from 8.0% to 27.1% (mean 15.6%). Two of these studies evaluated causes of death among childhood cancer survivors, while the remaining study focused on a specific and rare population (non-Hodgkin lymphoma with Nijmegen Breakage Syndrome). In terms of reporting of deaths, 11/62 (17.7%) therapeutic randomized trials and 2/5 (40.0%) TRM studies reported deaths before starting treatment. The table also illustrates the number of studies that report deaths occurring after completing treatment, after HSCT, and deaths from accidents, suicide and of unknown cause.

This systematic review demonstrates the absence of TRM definitions identified in studies of children, adolescents and young adults with lymphomas, solid tumors and brain tumors. Moreover, it highlights heterogeneity in reporting of deaths among these studies.

Although there is an overall lack of TRM definitions in pediatric cancer studies, TRM appears to be more often defined in pediatric acute leukemia trials than those of lymphomas, solid tumors and brain tumors. Our previous systematic review reported that 6.3% of pediatric acute lymphoblastic leukemia (ALL) and 66.7% of pediatric acute myeloid leukemia (AML) studies provided definitions for TRM, in contrast to the complete absence of TRM definitions in studies involving lymphoma, solid tumor and brain tumor patients [4]. Similarly, while early deaths was often used and defined in acute leukemia trials, this concept was only identified in one randomized trial of solid tumor patients [4]. In that particular study, the authors used a cut-off of 6 weeks from treatment initiation to define early death, a common time frame to delineate death as early in pediatric AML studies.

TRM represents an important outcome that may impact on patient management as well as therapeutic trial design. An example in pediatric oncology where TRM has been important is in children with Down syndrome and ALL (DS-ALL). The observation of excessive treatment-related deaths in these children from infections caused by chemotherapy-induced myelosuppression has led to treatment modifications specifically for children with DS-ALL [7–9]. These consisted of reducing therapy intensity in addition to enhancing supportive care (for example, leucovorin rescue, early empiric antibiotics, and admission during neutropenic episode) which has contributed to improving survival [10]. In contrast, children with DS and AML do not experience excess treatment-related death when treated with standard AML chemotherapy protocol developed for the general pediatric population but appear to have a favorable infectious profile when treated with DS-specific protocols for AML [11]. Knowing TRM rates in children with DS-ALL and DS-AML has lead to the adoption of specific strategies to improve outcome for both groups. Hence, defining and understanding TRM accurately serve as the foundation to adequately balance between therapy modification and supportive care implementation.

Another example to emphasize the importance of TRM can be appreciated from Head Start III (HS-III), a non-randomized, prospective, multi-institutional clinical trial evaluating the feasibility of an intensive induction followed by myeloablation with autologous hematopoietic stem cell rescue for young children with previously untreated malignant brain tumors. In January 2007, Regimen D of HS-III was suspended by the Data Safety Monitoring Committee pending review of the treatment-related deaths among children less than 18 months of age treated on this arm. Of the 19 patients, five died during induction from treatment toxicity. Causes of death were infections (n = 4), myocarditis (n = 1) and hemorrhage not related to disease (n = 1) [12]. In October 2007, the study reopened with reduction in chemotherapy doses in addition to more rigorous supportive care requirements. In light of the significance of TRM, there is an urgent need to develop a consistent definition of TRM for use across trials. Ideally, the same definition could be used for both hematologic and solid malignancies. Another important endeavor is to identify a consistent cause-of-death attribution system for TRM which will highlight the different distributions for deaths on and off treatment. However, determining the cause of death can represent a challenge due to the complexity of the disease and inter-individual interpretations. Therefore, a consensus approach will be adopted to develop a TRM algorithm that can be reliably operated across different abstractors, protocols, institutions and countries.

This review has unique strengths. To the best of our knowledge, it is the first to systematically evaluate and describe TRM definitions in studies of children, adolescents and young adults with lymphomas, solid tumors and brain tumors. Inclusion of studies from 1990 and forward captures more recent TRM definitions in more current trials and provides a more accurate estimate of TRM definition use. However, similar to other systematic reviews, it is limited by the methodological quality and outcome reporting of the included studies. Also, we did not include studies published in languages other than English. It is possible that these publications had a greater focus on TRM.

## Conclusions

In conclusion, this systematic review reports the absence of TRM definitions among studies of children, adolescents and young adults with lymphomas, solid tumors and brain tumors. As a better understanding of TRM is crucial in choosing specific strategies to improve survival of children with cancers, further work should prioritize the development of a consistent TRM definition that can be used across different diagnoses categories. A consensus approach is likely the best approach to create such a definition.

## Electronic supplementary material

Additional file 1: **Search strategies.** Strategies used to perform comprehensive searches for relevant trials. (DOCX 16 KB)

## Abbreviations

TRM: Treatment-related mortality
PRISMA: Preferred Reporting Items for Systematic reviews and Meta-Analysis
EBM: Evidence-Based Medicine
HSCT: Hematopoietic stem cell transplantation
MDS: Myelodysplastic syndrome
RCT: Randomized controlled trial
IRSG: International Rhabdomyosarcoma Study Group
ALL: Acute lymphoblastic leukemia
AML: Acute myeloid leukemia
DS: Down syndrome
HS-III: Head Start III.
