# Supplementary material for: Lack of treatment-related mortality definitions in clinical trials of children, adolescents and young adults with lymphomas, solid tumors and brain tumors: a systematic review
Source: BMC Cancer. 2014 Aug 26;14:612. doi: 10.1186/1471-2407-14-612 (PMC4152582; doi:10.1186/1471-2407-14-612)
Supplement: Supplementary file 1 — Additional file 1: Search strategies. Strategies used to perform comprehensive searches for relevant trials. (DOCX 16 KB) [file 12885_2014_4795_MOESM1_ESM.docx]

Additional file 1. Search strategies

OVID MEDLINE

Randomized anti-cancer trial

1 exp neoplasms/dt, ra, su, th [****Therapeutic subheadings****] (1013064)

2 exp neoplasms/ (2511544)

3 exp Antineoplastic Agents/ or exp Drug Therapy/ or exp Combined Modality Therapy/ or exp Surgical Procedures, Operative/ or radiotherapy setup errors/ or radiotherapy, adjuvant/ or exp radiotherapy, computer-assisted/ or exp radiotherapy dosage/ or exp radiotherapy, high-energy/ or radiotherapy, image-guided/ [****Theapeutic subject headings****] (3893922)

4 1 or (2 and 3) [****Base clinical set****] (1292662)

5 (randomized controlled trial or controlled clinical trial).pt. or randomized controlled trials/ or random allocation/ or double-blind method/ or single-blind method/ or ((singl* or doubl* or trebl* or tripl*) adj (mask* or blind*)).mp. [****RCT subject terms or textwords****] (618758)

6 4 and 5 [****Base clinical set study designs****] (54334)

7 limit 6 to "all child (0 to 18 years)" (6165)

8 (infan* or neonat* or child* or adolescen* or teen* or girl* or boy* or youth* or tot or tots or toddler* or paediatric* or pediatric*).mp. [***Age group Textword search terms***] (3131099)

9 7 or (6 and 8) [****Age group limits****] (6486)

10 limit 9 to yr="1980 -Current" [****Final Results Therapeutic studies****] (6185)

TRM

1 exp Neoplasms/ (2511544)

2 ((therapy or therapeutic or treatment or toxic or "non-relapse") adj3 (death* or mortality)).ti,ab. [****TRM Outcomes****] (19173)

3 11 and 12 [*****Base clinical set TRM outcomes****] (8165)

4 limit 13 to "all child (0 to 18 years)" (1611)

5 (infan* or neonat* or child* or adolescen* or teen* or girl* or boy* or youth* or tot or tots or toddler* or paediatric* or pediatric*).mp. [***Age group Textword search terms***] (3131099)

6 14 or (13 and 15) [****TRM Outcomes Age group limits****] (1695)

7 limit 16 to yr="1980 -Current" (1667)

EMBASE

Randomized anti-cancer trial

1 exp neoplasm/dt, rt, su, th [Drug Therapy, Radiotherapy, Surgery, Therapy] (1049497)

2 exp neoplasm/ (3492197)

3 xp antineoplastic agent/ or exp therapy/ or exp drug therapy/ or exp radiotherapy/ or exp cancer therapy/ or exp surgery/ [****Multimodal combined therapy is included in the cancer therapy subject heading; Cancer surgery and Surgical Procedures, Operative are included under Exp Surgery****] (8145294)

4 1 or (2 and 3) [****Base clinical set****] (1825612)

5 ct.fs. or controlled clinical trial/ or randomized controlled trial/ or randomization/ or double blind procedure/ or single blind procedure/ or triple blind procedure/ or (((singl* or doubl* or trebl* or tripl*) adj (mask* or blind*)) or rct or rcts).mp. [****RCT subject terms or textwords****] (897365)

6 4 and 5 [*****Base clinical set study designs****] (155221)

7 limit 6 to (infant <to one year> or child <unspecified age> or preschool child <1 to 6 years> or school child <7 to 12 years> or adolescent <13 to 17 years>) (7493)

8 (infan* or neonat* or child* or adolescen* or teen* or girl* or boy* or youth* or tot or tots or toddler* or paediatric* or pediatric*).mp. [***Age group Textword search terms***] (3370896)

9 7 or (6 and 8) [****Age group limits****] (11193)

10 limit 9 to yr="1980 -Current" [****Final Results Therapeutic studies****] (11088)

TRM

1 exp Neoplasms/ (3492197)

2 ((therapy or therapeutic or treatment or toxic or "non-relapse") adj3 (death* or mortality)).ti,ab. (28785)

3 11 and 12 (12330)

4 limit 13 to (infant <to one year> or child <unspecified age> or preschool child <1 to 6 years> or school child <7 to 12 years> or adolescent <13 to 17 years>) (1546)

5 (infan* or neonat* or child* or adolescen* or teen* or girl* or boy* or youth* or tot or tots or toddler* or paediatric* or pediatric*).mp. (3370896)

6 13 and 15 (1826)

7 14 or 16 (1826)

8 limit 17 to yr="1980 -Current" (1786)

EBM Reviews - Cochrane Central Register of Controlled Trials

Randomized anti-cancer trial

1 exp neoplasms/dt, ra, su, th [****Therapeutic subheadings****] (11117)

2 exp neoplasms/ (37860)

3 exp Antineoplastic Agents/ or exp Drug Therapy/ or exp Combined Modality Therapy/ or exp Surgical Procedures, Operative/ or radiotherapy setup errors/ or radiotherapy, adjuvant/ or exp radiotherapy, computer-assisted/ or exp radiotherapy dosage/ or exp radiotherapy, high-energy/ or radiotherapy, image-guided/ [****Theapeutic subject headings****] (174708)

4 1 or (2 and 3) [****Base clinical set****] (29952)

5 (randomized controlled trial or controlled clinical trial).pt. or randomized controlled trials/ or random allocation/ or double-blind method/ or single-blind method/ or ((singl* or doubl* or trebl* or tripl*) adj (mask* or blind*)).mp. [****RCT subject terms or textwords****] (435011)

6 4 and 5 [****Base clinical set study designs****] (29948)

7 adolescent/ or exp child/ or exp infant/ (98340)

8 (infan* or neonat* or child* or adolescen* or teen* or girl* or boy* or youth* or tot or tots or toddler* or paediatric* or pediatric*).mp. [***Age group Textword search terms***] (131959)

9 6 and (7 or 8) (4641)

10 limit 9 to yr="1980 -Current" (4369)

TRM

1 exp Neoplasms/ (37860)

2 ((therapy or therapeutic or treatment or toxic or "non-relapse") adj3 (death* or mortality)).ti,ab. [****TRM Outcomes****] (2815)

3 11 and 12 [*****Base clinical set TRM outcomes****] (816)

4 adolescent/ or exp child/ or exp infant/ (98340)

5 (infan* or neonat* or child* or adolescen* or teen* or girl* or boy* or youth* or tot or tots or toddler* or paediatric* or pediatric*).mp. [***Age group Textword search terms***] (131959)

6 13 and (14 or 15) (157)

7 limit 16 to yr="1980 -Current" (154)
